# Evaluation of the Virulence and Plant Growth-Promoting Potential of Endophytic Bacteria for Improving Vegetable Production

**DOI:** 10.1007/s00284-025-04356-1

**Published:** 2025-07-10

**Authors:** Adekunle Raimi, Suranie Horn, Rialet Pieters, Rasheed Adeleke

**Affiliations:** 1 Unit for Environmental Sciences and Management, North-West University, Potchefstroom, 2520 South Africa; 2 Occupational Hygiene and Health Research Initiative (OHHRI), Faculty of Health Science, North-West University, Private Bag X6001, Potchefstroom, South Africa

## Abstract

Beneficial microbes, including endophytic bacteria, are widely used in bioformulation to improve crop productivity. However, the microbes are rarely assessed for their virulence properties. Thus, this study evaluates bacterial endophytes from vegetables for their hemolytic, antibiotic, cytotoxic activity, and ability to colonise and promote spinach (*Spinacia oleracea*) growth. Colonisation was assessed by visualising mCherry plasmid-tagged endophytic bacteria within plant tissues using a confocal microscope. Results showed that endophytic *Pseudomonas azotoformans, Enterobacter bugandensis, Bacillus cereus* and *Serratia marcescens* were non-hemolytic, sensitive to antibiotics, with high antibacterial and very slow cytotoxic activity, suggesting their nonvirulence and biocontrol potential. These bacteria had the highest and fastest rate of promoting seed germination and colonised the spinach leaves in a greenhouse experiment, as revealed by the visualised red fluorescence of the mCherry-plasmid transformed endophytes. Spinach inoculated with *E. bugandensis* and *S. marcescens* had higher total chlorophyll, but lower growth parameters compared to *P*. *azotoformans* and *B. cereus.* The root length, fresh weight and dry weight were higher in plants inoculated with* P*. *azotoformans* compared to the control and other endophytes. While endophytes with virulence potential may present health implications when consumed by humans, the nonvirulent and plant growth-promoting endophytes have applications in microbial formulation. Overall, beneficial endophytes present valuable resources in inoculum production for sustainable crop production.

**Supplementary Information:**

The online version contains supplementary material available at 10.1007/s00284-025-04356-1.

## Introduction

Over the years, biotechnologists have extensively studied and harnessed plant–microbe interactions to improve plant health, productivity, and nutritional quality. The plant–microbe interaction is beneficial to both parties, with considerable impact on host plant growth and health, while the host plant provides a safe niche and chemical substances for the microbes [1]. A unique group of microbes inhabiting plant tissues without causing harm to the host are known as endophytes, contributing to global element cycling, nutrient solubilisation, enzyme production and plant stress tolerance [2]. They also produce plant growth hormones, such as auxin, ethylene, and cytokinin [3], mobilise soil nutrients, e.g., phosphate, fix nitrogen and synthesize substances that are biocontrol agents [2]. Thus, endophytes are viable plant growth-promoting (PGP) agents with huge applications in agroecosystems as an inexpensive, eco-friendly, and sustainable technology.

Endophytes are a better resource in inoculant technology compared to non-endophytes due to their compatibility, reinfection and colonisation capability with the host plants, and the complexity of their interactions, which have huge ecological benefits [3]. Previous studies have investigated the PGP ability of inoculants of endophytes on economically important crops such as maize, sunflower, and wheat [4, 5]. However, despite the rising demand and health benefits of vegetable crops, they are less investigated for their beneficial endophytes, consequently presenting a research gap that needs to be addressed to improve sustainable vegetable production.

Leafy vegetables are an important part of the human diet, mostly eaten raw as salad without any form of thermal processing that can alter their microbiological quality. Vegetables like spinach, carrot, beetroot, cabbage, lettuce, and onion have various human health benefits, including a reduction in obesity and high blood pressure due to their high nutrient density [6]. However, the vegetable endosphere is regarded as a reservoir for emerging opportunistic pathogens [7], suggesting that the assessment of their microbiological quality should be a priority. Interestingly, vegetables have been implicated in foodborne disease outbreaks in many parts of the world. For example, *Listeria monocytogenes*, a major threat to the food industry*,* has been reported in South Africa and the United States [8, 9]. This pathogen is the causative agent of several outbreaks of listeriosis worldwide and has been reported as an endophyte of vegetable crops [10]. According to Berg et al. [11], bacterial endophytes, including *Pseudomonas*, *Enterobacter*, *Acinetobacter*, *Staphylococcus*, *Serratia* and *Bacillus* have bivalent associations with the host plant, contributing to PGP and biocontrol of pathogens and causing diverse diseases when they colonise human organs. Therefore, it is crucial to assess the pathogenicity and virulence potential of endophytes to gain full insight into their importance in agroecosystems and human health management.

This study aimed to investigate the PGP ability of bacterial endophytes of vegetables in a pot experiment. It further assessed the antibacterial, antibiotic susceptibility and virulence potential of bacterial endophytes using selected pathogens, antibiotics, and cytotoxic activities on the HuTu-80 human intestinal cell line. The study hypothesised that bacterial endophytes could colonise plant tissues with differential effects on seed germination rate and plant growth. To confirm successful colonisation, a confocal microscope was used to visualize the bacterial endophytes that colonise the plant tissues. Confocal microscope systems in combination with the fluorescence protein gene have been used in plant–microbe interaction studies to determine the ecological niche of endophytes after inhabiting the host plants [12]. This study may provide insights into the selection criteria for efficient microbial inoculum and how they can be used to improve vegetable productivity without any adverse effects on human health. In addition, it may unravel the potential of leafy vegetables to spread antibiotic-resistant bacteria, thereby improving the characteristics profile of the investigated endophytes, which is crucial for making key decisions in sustainable agriculture and biotechnological applications.

## Materials and Methods

### Bacterial Endophyte Culture

The isolation and screening of PGP bacterial endophytes used for this experiment have been reported in a previous study [2]. Based on the PGP attributes and 16S rRNA gene sequencing for bacterial identification, efficient PGP bacterial endophytes with the potential for N-fixation, phosphorus solubilisation and production of siderophores, hydrogen cyanide, and 1-aminocyclopropane-1-carboxylic acid (ACC) deaminase were selected for further study. The present study further assessed the bacterial endophytes’ virulence potential, antibiotic susceptibility, cytotoxicity, and in vivo PGP capability.

The selected beneficial bacterial endophytes and test organisms, including *Enterococcus faecalis*, *Escherichia coli*, *Klebsiella pneumoniae*, *Micrococcus luteus* and *Serratia marcescens* were freshly grown on nutrient agar plates for 24 h. Pure colonies were inoculated into a Mueller–Hinton broth (MHB) (Biolab, CA, USA) and incubated overnight at 36 ℃ with shaking at 100 rpm. The growth was stopped during the log phase. The freshly grown culture was adjusted to 0.5 McFarland standard (1.5 × 10^8^) or the turbidity was adjusted to an optical density (OD) of 0.1 with MHB using a spectrophotometer at 600 nm. These cultures were used for the antibacterial, antibiotic susceptibility and cytotoxicity tests.

### Hemolytic Activity, Antibiotic Susceptibility, Crude Extract Production and Minimum Inhibitory concentRation (MIC) Assessment

Hemolytic activity was assessed using 5% sheep blood agar (Biolab, MA, USA) incubated at 37 ℃. A clear zone or green colouration around the colonies indicates beta and alpha hemolytic activities, respectively. The DNase agar (Merck, South Africa) containing pancreatic digest of casein 15 g, papaic digest of soybean meal 5 g, sodium chloride 5 g, deoxyribonucleic acid 2 g and agar 15 g, pH 7.3 ± 0.2 at 25 ℃ was used for the enzyme production test. The plates were flooded with 1 N HCl and a clear zone around the colonies or streak is positive, while no colour change is a negative result. The Kirby-Bauer disk diffusion method was employed for the antibiotic-susceptibility testing following the protocol described by Hudzicki [13]. Four antibiotic classes were used based on the mechanisms of action (Supplementary Table 1). Antibiotic disks, including chloramphenicol (30 µg/disc), gentamicin (10 µg/disc), ampicillin (10 µg/disc), vancomycin (30 µg/disc), tetracycline (30 µg/disc), streptomycin (30 µg/disc), ciprofloxacin (5 µg/disc), cefazolin (30 µg/disc), trimethoprim (10 µg/disc), erythromycin (15 µg/disc), amoxicillin (20 µg/disc), clindamycin (2 µg/disc) of 6 mm diameter (Biolab, South Africa) were placed on Mueller–Hinton agar plates spread inoculated with the bacterial endophytes for 24 h at 37 ℃. Inhibition zones were measured and compared with clinical and laboratory standard institute (CLSI) standards to classify the isolates as susceptible, intermediate, or resistant to antibiotics. The crude extract of selected bacterial endophytes was made, and the minimum inhibitory concentration (MIC) was measured following the method of Kumar et al. [14], as explained in Supplementary Text 1. A 20 mg bacterial extract was dissolved in 1 mL of dimethyl sulfoxide to make a stock solution of 20 mg/mL, which was diluted using Mueller–Hinton broth as the diluent to obtain different dilutions of 128 to 0.5 mg/mL. The MIC value is the lowest concentration of the compound showing no visible growth.

### Cytotoxicity Assay Using the xCELLigence Cell Analyser System

HuTu-80 human intestinal cells (HTB-40™) obtained from the American Type Culture Collection (ATCC) (Manassas, VA, USA) were maintained as described in Supplementary Text 2. The cytotoxic potential of endophytic bacteria on human intestinal cell line was determined using a real-time cell analyser, the xCELLigence system RTCA single plate instrument (ACEA Biosciences Inc., San Diego, CA, USA) with RTCA software (version 1.2.1). The software records the electrical impedance that flows across micro-electrodes at the bottom of each well of the gold-plated E-plate. The growth of Hutu-80 cells is measured in real-time and converted into cell index (CI) values, expressed in terms of the CI of the unexposed control cells (receiving DMEM only) and the percentage viability was calculated. Cells were seeded at a density of 8.0 × 10^4^ cells/ml and left to adhere for 24 h. The cell line was exposed to 10 µL of different endophytic bacterial cells (1.5 × 10^8^) solutions in triplicate. Three wells with cells that received DMEM culture medium were the control. Impedance readings were recorded every 10 min for 186 h. The cytotoxicity effects were calculated based on the time taken for the treated HuTu-80 cells to significantly decrease from the untreated control. Isolates that caused significant cytotoxic effects within shorter periods were considered more virulent [15].

### Seed Germination Test

This study used spinach because it is easy to cultivate and grows to maturity within a short period. Spinach is well cultivated using the greenhouse and requires less technicalities or expertise. Seeds were procured from Die Blomwinkeltjie Florist (Pty) Ltd., Potchefstroom, South Africa, and seed viability was assessed as described by Davies et al. [16]. Of the different techniques available for confirming seed viability, germination testing is the most accurate and reliable as described in Supplementary Text 3. The following equations were used for calculating the percentage of germination and viability (Davies et al. [16]).

1 $$Germination \left( \% \right) = \frac{g}{x} * 100$$

2 $$Viability \left( \% \right) = \frac{{\left( {g + f + a} \right)}}{x} * 100$$

where: g = numbers of germinated seeds, x = numbers of sown seeds (infested and empty seeds are removed), f = no of fresh seeds and abnormal seedlings. a = no of abnormal seedlings.

The surface-sterilised seeds were soaked in freshly prepared bacterial inoculum (1.5 × 10^8^ CFU/mL) in a beaker for about 24 h before determining the germination and growth rate following the method described by Makhaye et al. [17]. Seeds in sterilised distilled water were used as a control. Parameters including mean germination time (MGT), germination index (GI), germination rate index (GRI), first day of germination (FDG), final germination percentage (FGP), coefficient of velocity of germination (CVG), and time spread of germination (TSG), were used to determine the germination dynamics. FGP (%) is the final number of seeds that germinated in percentage.

3 $$MGT = \Sigma f.x/\Sigma f$$

4 $$CVG = N1 + N2 + \cdot \cdot \cdot + Nx/100 \times N1T1 + \cdot \cdot \cdot + NxTx GRI$$

5 $$GRI = G1/1 + G2/2 + \cdot \cdot \cdot + Gx/x$$

6 $$GI = \left( {10 \times n1} \right) + \left( {9 \times n2} \right) + \cdot \cdot \cdot + \left( {1 \times n10} \right)$$

where: f = seeds germinated on day x, N = number of seeds germinated each day, T = number of days from seeding corresponding to N, G1 = germination percentage on the first day after sowing, G2 = germination percentage on the second day after sowing, n1, n2... n10 = numbers of germinated seeds on the 1 st, 2nd until the 10th day; and. 1 = weights given to the number of germinated seeds on the 1 st, 2nd, and subsequent days.

### Endophytic Bacterial Cell Transformation with mCherry Insert Plasmids

Plasmid pLV-mCherry (Addgene plasmid #36084), containing a 6782 base-pair backbone and a 704 base-pair gene insert, was acquired from the Addgene repository in 2022. The plasmid expresses red fluorescence due to its gene insert encoding monomeric red fluorescent proteins with antibiotic-resistant genes for ampicillin used as selection markers. A single bacterial colony containing the plasmids were first grown in 15-ml tubes containing 2 mL LB broth (Biolab Diagnostics, South Africa) supplemented with ampicillin (100 µg/mL) for 24 h at 100 rpm and 37 °C in a shaker incubator. Using the ZymoPURE™ Plasmid Miniprep kit (Zymo Research, CA, USA), the plasmid DNA was extracted from the bacteria and diagnostic restriction digestion was performed with *Hind*III, *Nde*I, *Xba*l and *Sal*I (New England Biolabs, Germany). The plasmid’s integrity and quality were verified on a 1.5% agarose gel electrophoresis and visualised in a Gel Doc (BioRad Laboratories, CA, USA).

#### Screening and Verification of Transformed Bacterial Cells

The endophytic bacteria were first made into competent cells and transformed with mCherry following the protocol described by Bjornson et al. [18] as explained in Supplementary Text 4. After adding an equal volume of 40% glycerol, the transformed endophytic bacteria were stored at − 80 °C. The transformed bacterial cells were screened using their ability to grow on freshly prepared LB agar supplemented with 100 µg/mL of ampicillin, on which the untransformed bacterial cells could not grow. The cells were screened for fluorescence expression using the confocal imaging systems (ZEISS Microscopy, Deutschland, Germany), reconstructed with Zeiss LSM 980 with Airyscan 2 and equipped with C-PlanApochromat 63 ×/1.4 Oil DIC UV–Vis IR M27 objective with 32-channel detectors. Successful fluorescence gene incorporated into the bacterial cells was verified using the total genomic DNA extracted from a washed colony pellet transformants using ZymoBIOMICS™ DNA Miniprep Kit (Zymo Research, CA, USA). Primer sequences (mCherry-F, 5′-CCCCGTAATGCAGAAGAAGA-3′; mCherry-R, 3′-TTGGTCACCTTCA GCTTGG-5′) that amplify the mCherry insert were utilised in a polymerase chain reaction [19]. The PCR consist of a 25 µL reaction comprising 12.5 µL of one Taq 2 × Master Mix with standard buffer (New England Biolabs Inc., CA, USA), 10 µM each of forward and reverse primers, ~ 50 ng plasmid DNA template and PCR-grade water to final volume. The PCR conditions were initial denaturation at 95 °C for 5 min, followed by 35 cycles of 95 °C for 30 s, 55 °C for 30 s and 72 °C and a final extension at 72 °C for 5 min [19].

### Pot Trial and Bacterial Endophyte Colonisation and localisation Assessment

The pot experiment used two spinach (*Spinacia oleracea* L.) variant types [BeetFordhook Giant (SBG)] and [Beet Lucullus (SBL)] to study the growth-promoting ability of the mCherry-tagged endophytes. Pool sand (0.5–1.2 mm size), vermiculite and plastic pots (4 L) were sterilised before use in the greenhouse. The soil was mixed with the vermiculite in a ratio of 3:1 to increase the soil moisture-retaining capacity. The greenhouse was maintained at a temperature of 18–21 °C during the day and 10–12 °C at night, a relative humidity of 45–50% and 12–14 h of light per day. Seeds were sown at a depth of 10 mm with 7 × 30 cm spacing, and ten inoculated seeds were sown in each of the pots filled with sterile soil. The soil was kept consistently moist by regular watering without being waterlogged. Each of the four selected beneficial bacterial endophytes represents a treatment (T1, T2, T3, and T4) while the uninoculated seeds treatment (T0) is the control. The experiment was made in quadruplicate and arranged in a randomized complete block design (RCBD). The transformed and untransformed beneficial bacterial endophytes were compared to ascertain the impact of transformation on PGP ability. The vigour index and disease incidences were also evaluated using the formula below [20].

$${\text{Vigour index }} = \, \left[ {\left( {{\text{mean root length }}\left( {{\text{cm}}} \right) \, + {\text{ mean shoot length }}\left( {{\text{cm}}} \right)} \right) \, \times {\text{ percentage of seed germination}}} \right]$$

Shreelalitha et al. [20]

$${\text{Disease incidence }} = \, \left( {{\text{No of infected plants }}/{\text{ No of total plants}}} \right) \, \times { 1}00$$

Gashew et al. [21]

#### Evaluation of Chlorophyll Content, Vigour Index and Fresh and Dry Weight

The seed germination percentage and vigour index were evaluated for each treatment after 20 days, following the method of Shreelalitha et al. [20]. The plants were harvested after two months of growth, and yield parameters, including the plant height, root length, number of leaves, plant fresh weight, and dry weight, were measured. To determine the dry weight, plant samples were cut into pieces, placed in a crucible and oven-dried at 65 °C for 2 days until a constant weight was achieved. The chlorophyll a and b content were quantified using the methods of Pérez-Patricio et al. [22], as described in Supplementary Text 5.

#### Visualisation of Red Fluorescence Protein-Tagged Endophytic Bacterial Cells in Spinach Tissues

Microscopic observations of bacterial transformants and the colonised plant tissues were captured using a confocal microscope (ZEISS Microscopy, Deutschland, Germany). The plant tissues were cut into pieces (10 mm × 10 mm), fixed on a microscopic slide using Eukitt Quick Mounting Media (Thermo Scientific, USA) and allowed to stand for 2 h before visualization. Bacterial cells were excited at 594 nm, and emission was detected between 587 and 610 nm. Autofluorescence within the leaves were excited with the 405 nm diode laser and the emission was detected between 436 and 484 nm.

### Data Analysis

The cytotoxicity data was normalised using the RTCA software (version 1.2.1). Normalisation was done by manipulating data at a specific time point (19 h) (addition of endophytic bacterial cells), which is set as 1.0 by the software, comparing the cytotoxicity index (CI) value of the control to the CI of the treated cells. The SPSS statistical package version 29 (IBM) was used to determine the time point at which the response of the treated cells was statistically significantly (*P* < 0.05) different from the untreated cells. Vegetable yield and germination data were analysed for normality using the Shapiro–Wilk test and if data were not normally distributed and could not be normalized using log or square root transformation, nonparametric tests such as the Mann–Whitney U test and Kruskal–Wallis test were used, otherwise, parametric tests such as two-sample t-test and ANOVA were applied and significant value was considered at *P* < 0.05. For significant values, the post hoc analysis of the means was performed with Tukey’s multiple comparisons.

The 16S rRNA sequences for the isolates used in this study have been deposited in the GenBank database of the National Centre for Biotechnology Information, with accession numbers OL634983-OL635015, OL860938-OL860940 and OL897282-OL897284 and PQ681303-PQ681309 [2]. 

## Results

### Hemolysis and DNase Enzyme Activity

Most of the bacterial endophytes tested for virulence properties, only 15% and 26% of bacterial endophytes were beta and alpha-hemolytic, respectively, indicating that the bacteria participate in the lysis and damage of red blood cells. Most of the endophytes are gamma hemolytic (59%), which cannot lyse the red blood cells. The genus *Bacillus* had the highest percentage (22%) of endophytes exhibiting gamma hemolysis followed by *Pseudomonas* (15%). For DNase production, about 33% of the endophytes were positive, which indicates that the bacteria could hydrolyse deoxyribonucleic acid. About 29% and 25% of *Bacillus* and *Pseudomonas* tested were positive for DNase production, respectively. Three of the bacterial endophytes: *Staphylococcus cohnii*, *Priesta megaterium*, and *Neobacillus bataviensis* were positive for both hemolysis and DNase enzyme activity. 

### Antibiotic Sensitivity of Bacterial Endophytes and Antibacterial Properties of Crude Extracts

Of the 16 bacterial endophytes tested for antibiotic susceptibility, three were resistant to at least one of the four classes of antibiotics tested (Table 1) as revealed by the zone of inhibition. Eight bacterial endophytes were either susceptible or intermediate, while *P. azotoformans*, *E. bugandensis*, *B. cereus*, *P. agglomerans*, and* S. marcescens* were susceptible to most of the antibiotics. All the endophytes were found to be susceptible to streptomycin. About 44% of the endophytes were resistant to amoxicillin, while 69% exhibited intermediate sensitivity to ciprofloxacin (Table 1). All the bacterial endophytes produced active extracts that inhibited at least one of the test organisms. The MIC of the extracts from *B. cereus* against *S. marcescens* and *E. faecalis* was 0.5 mg/mL, while the extracts from *P. azotoformans* had a MIC of 2 mg/mL against *K. pneumonia*. In addition, the crude extract from *P. agglomerans* and *E. bugandensis* had a MIC of 0.5 mg/mL against *M. luteus*.

**Table 1:** Antibiotic sensitivity pattern of bacterial endophytes recovered from vegetable crops

Organism	Cell wall biosynthesis				Protein synthesis						Folic acid	DNA synthesis	
	Amp	Van	Cef	Amo	Gen	Tet	Stre	Chlo	Ery	Cli	Tri	Cip	Class
*Pseudomonas azotoformans* OL634986	S	I	S	S	S	I	S	I	S	S	S	I	0
*Pantoea agglomerans* OL635006	S	S	S	S	S	I	S	I	S	S	S	I	0
*Bacillus haynesii* OL897283	R	I	R	R	S	R	S	R	S	S	R	R	4
*Bacillus cereus* OL634993	S	S	S	S	S	I	S	I	S	S	S	I	0
*Bacillus mycoides* OL634994	R	I	R	R	S	R	S	R	I	I	R	R	4
*Stenotrophomonas maltophilia* OL635015	R	I	S	R	S	I	S	I	S	S	S	I	1
*Comamonas terrigena* OL634999	S	I	R	R	R	I	S	I	S	I	R	R	4
*Paenibacillus amylolyticus* PQ681303	S	S	S	S	S	S	S	I	S	I	S	I	0
*Priesta megaterium* OL860938	S	I	S	R	I	S	S	I	S	S	S	I	1
*Leclercia adecarboxylata* OL635004	S	S	S	S	S	S	S	S	S	S	S	I	0
*Bacillus pumilus* OL634996	R	I	S	R	S	I	S	S	S	R	R	S	3
*Bacillus subtilis* OL897282	S	I	S	S	S	S	S	I	S	I	R	I	1
*Serratia marcescens* PQ681309	S	S	S	S	S	I	S	S	R	R	S	I	2
*Acinetobacter haemolyticus* OL634983	R	I	R	R	S	I	S	I	S	S	R	I	2
*Microbacterium paraoxydans* OL634985	I	S	S	S	S	R	S	S	S	S	S	S	2
*Enterobacter bugandensis* OL635000	S	S	S	S	S	I	S	I	S	S	S	I	0

Degree of susceptibility: > 20 mm—Sensitive; 10–19.9 mm—intermediate; 0.0–9.9 mm resistant. chlo, Chloramphenicol (30 µg/disc); Gen, Gentamicin (10 µg/disc); Amp, Ampicillin (10 µg/disc); Van, Vancomycin (30 µg/disc); Tet, Tetracycline (30 µg/disc); Stre, Streptomycin (30 µg/disc); Cip, Ciprofloxacin (5 µg/disc); Cef, Cefazolin (30 µg/disc); Tri, Trimethoprim (10 µg/disc); Ery, Erythromycin (15 µg/disc); Amo, Amoxicillin (20 µg/disc); Cli, Clindamycin (2 µg/disc). The antibiotics were classified according to their mechanisms of action

### Bacterial Endophytes Exhibit Different Levels of Cytotoxicity Test

The bacterial endophytes were grouped into the following categories: (A) Extremely high; (B) High; (C) Medium and (D) Slow, based on the time it took for them to cause statistically significant (*P* < 0.05) cytotoxicity of HuTu-80 cells compared to the untreated control cells (Table 2). Most of the endophytes (32%) significantly (*P* < 0.05) reduced the viability of the HuTu-80 cells within the shortest time, falling into the extremely high cytotoxicity category (Table 2). These endophytes, including *Bacillus mycoides, B. subtilis, Acinetobacter haemolyticus, Pseudomonas atacamensis* and *Cytobacillus solani*, caused significant cytotoxicity within 0.17–1 h. The high cytotoxic endophytes such as *Pseudomonas syringae, Pseudomonas agarici, Paenibacillus amylolyticus,* and *Rhizobium zeae* killed the HuTu-80 cells within 2–5 h when compared to the untreated control cells. The endophytes with medium cytotoxicity such *as Bacillus nakamurai, Neobacillus bataviensis* and *Comamonas terrigena* caused significant cytotoxicity of the HuTu-80 cells between 5 and 12 h. *B.* cereus, B. *haynesii, P. azotoformans*, *S. marcescens*, and *E. bugandensis* had a slow cytotoxicity effect, killing the HuTu-80 cells slowly and only after 12 h (Table 2). Another group of endophytes: *Lysinibacillus pakistanensis, Pantoea agglomerans* and *Microvirga calopogonii* had a cytotoxicity that could be described as gradual, killing the cells over a 20 h period compared to the control (Supplementary Fig. 1).

**Table 2:** Endophytic bacterial cytotoxicity and the significant (*P* < 0.05) killing time

Bacterial endophytes	Accession no	Time to cause significant cytotoxicity of HuTu-80 cells (h)	Category
*Bacillus subtilis*	OL897282	0.17	
*Acinetobacter haemolyticus*	OL634983	0.17	
*Pseudomonas atacamensis*	OL635011	0.17	
*Cytobacillus solani*	PQ681304	0.17	Extremely high
*Bacillus mycoides*	OL634994	0.17	
*Microbacterium paraoxydans*	OL634985	0.50	
*Bacillus pumilus*	OL634996	0.50	
*Priesta megaterium*	OL860938	1.00	
*Paenibacillus amylolyticus*	PQ681303	1.67	
*Staphylococcus cohnii*	OL635014	1.29	
*Pseudomonas syringae*	OL635012	1.59	High
*Pseudomonas agarici*	OL635010	2.00	
*Rhizobium zeae*	PQ681308	2.33	
*Enterobacter cloacae*	PQ681306	4.67	
*Bacillus nakamurai*	OL634995	5.00	
*Neobacillus bataviensis*	PQ681307	6.50	
*Xanthomonas fragariae*	OL634992	8.01	Medium
*Stenotrophomonas maltophilia*	OL635015	10.34	
*Comamonas terrigena*	OL634999	10.68	
*Pseudomonas azotoformans*	OL634986	12.35	
*Bacillus cereus*	OL634993	12.68	
*Enterobacter bugandensis*	OL635000	12.85	Slow
*Serratia marcescens*	OL634991	12.99	
*Bacillus haynesii*	OL897283	14.99	
*Pseudomonas gessardii*	OL634987	27.19	

### Bacterial Endophytes Improve Seed Germination

Eight endophytes, including *B.* cereus, *P. azotoformans*, *S. marcescens*, *E. bugandensis,* and *Pantoea agglomerans* were selected for seed germination testing due to their susceptibility to most of the tested antibiotics (Table 1) and very slow cytotoxic activity (Table 2). *Bacillus haynesii,* with slow cytotoxicity and, *Pseudomonas atacamensis* and *Acinetobacter haemolyticus,* with high plant growth-promoting ability already reported in a previous study [2] were randomly selected. The results reveal a high germination rate for *Bacillus cereus* (treatment 1, T1), *Pseudomonas azotoformans* (treatment 2, T2), *Enterobacter bugandensis* (treatment 3, T3), and *Serratia marcescens* (treatment 4, T4), with over 70% mean germination percentage for the seeds. In addition, these bacterial endophytes influenced the seed germination parameters, including the final germination percentage, mean germination time, coefficient of velocity of germination, germination index, and germination rate index (Table 3). *P. azotoformans* had the highest and fastest rate of seed germination and germination rate per day, followed by *E. bugandensis*, *S. marcescens* and *B. cereus*, respectively. These bacteria promoted a high germination rate and were further used in the pot experiment. For *P*. *azotoformans*, about 98% of seeds germinated within the first 6 days, while 95% was observed for *S. marcescens.* Aside from *E. bugandensis*, which germinated on the 4th day, bacterial endophytes having above 70% GI had an FDG of 3 (Table 3). The CVG, which shows the speed of germination did not follow the same trend, it was highest in *E. bugandensis,* while *S. marcescens* and* P*. *azotoformans* had 1.15 and 1.12, respectively (Table 3).

**Table 3:** Effect of bacterial endophyte inoculants on seed germination parameters

Sample	FGP (%)	MGT (day)	GI	CVG	GRI	FDG
Control	75 ± 1.0 c	8.04 ± 0.96a	44.3 ± 2.0 g	1.21 ± 0.01a	75 ± 2.0d	7 ± 1.0a
*Pseudomonas atacamensis*	65 ± 3.0d	6.44 ± 0.48 cd	59.3 ± 1.5e	0.84 ± 0.01 g	65 ± 1.0e	3 ± 0.0c
*Acinetobacter haemolyticus*	55 ± 4.0f	6.64 ± 0.11 cd	48 ± 1.5f	0.73 ± 0.00i	55 ± 1.0 g	4 ± 1.0b
*Bacillus cereus*	85 ± 1.0b	6.42 ± 0.50cde	78 ± 2.0c	1.09 ± 0.02e	85 ± 3.0c	3 ± 0.0c
*Pantoea agglomerans*	50 ± 0.0 g	7.77 ± 0.11ab	32 ± 0,6i	0.78 ± 0.01 h	50 ± 1.5 h	7 ± 1.0a
*Pseudomonas azotoformans*	98 ± 2.0a	5.68 ± 0.21e	105 ± 3.0a	1.12 ± 0.01d	98 ± 1.6a	3 ± 0.0c
*Enterobacter bugandensis*	85 ± 2.0b	6.88 ± 0.44bc	70 ± 1.9d	1.17 ± 0.01b	85 ± 1.6c	4 ± 0.0b
*Serratia marcescens*	95 ± 3.0a	6.07 ± 0.33de	94 ± 2.0b	1.15 ± 0.02c	95 ± 2.5b	3 ± 0.0c
*Bacillus haynesii*	60 ± 1.0e	8.03 ± 0.47a	36 ± 0.0 h	0.96 ± 0.00f	60 ± 1.0f	6 ± 1.0a

The data are presented as mean ± standard deviation (*n* = 4). The main effect of the Group (inoculum treatments) is statistically significant (*P* < 0.05) and large. *FGP* final germination percentage, *MGT* mean germination time, *GI* germination index, *CVG* coefficient of velocity of germination and GRI germination rate index

### Microscopic Observation of Transformed Bacterial Endophytes and Colonised Plant Tissues

**Fig. 1:**
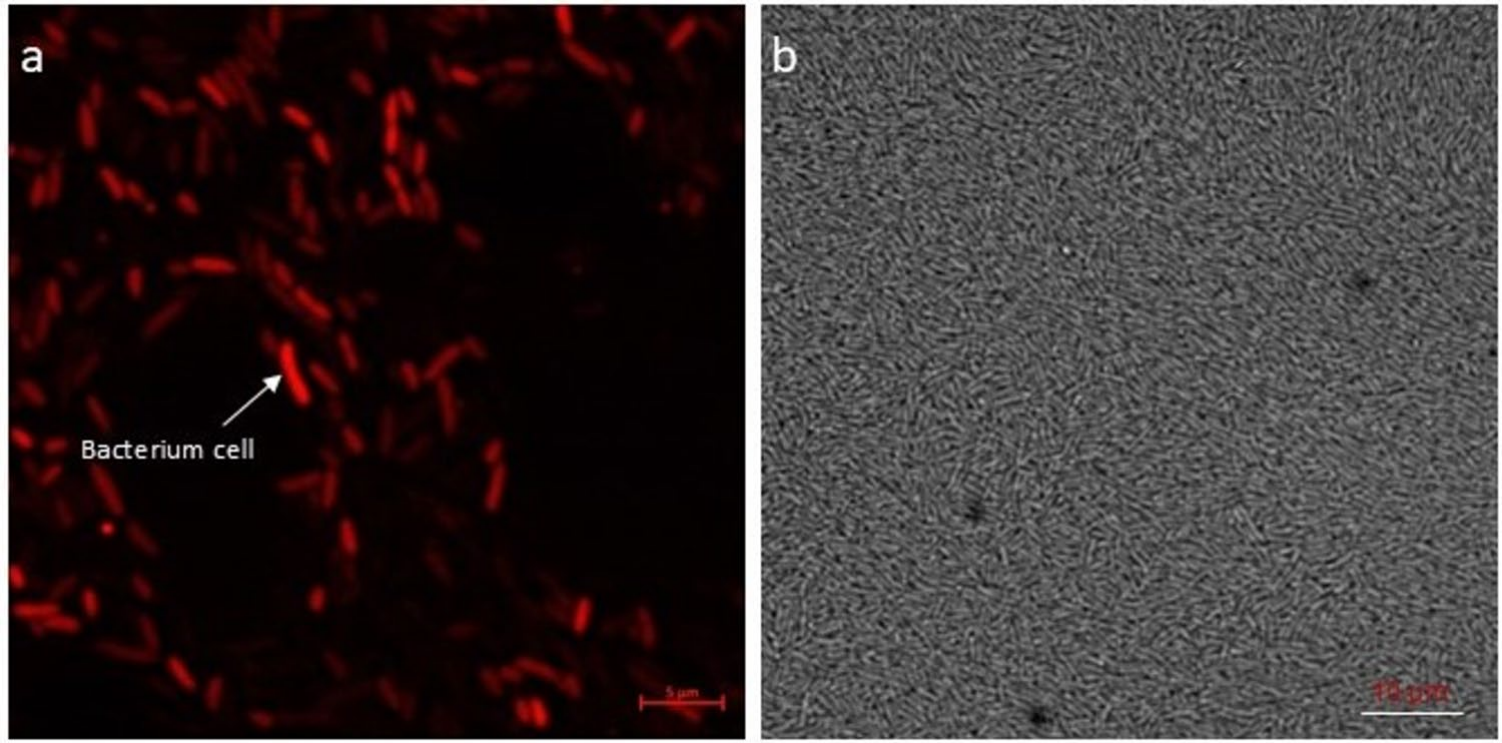
Confocal microscopy image showing **a** bacterial endophytes with red fluorescent protein pLV-mCherry plasmid and **b** bacterial endophytes without red fluorescent protein mCherry plasmid. The bacterial culture was grown in Lauria-Bertani medium for 24 h before microscopy examination

The bacterial transformants were visualized with a confocal microscope and red fluorescence signals were detected, indicating a successful transformation of the RFP-tagged bacterial transformants (Fig. 1). The red fluorescence expression was detected in the bacterial cells at an excitation wavelength of 587 nm and emission wavelength of 610 nm, while the untransformed control cells did not show fluorescence.

**Fig. 2:**
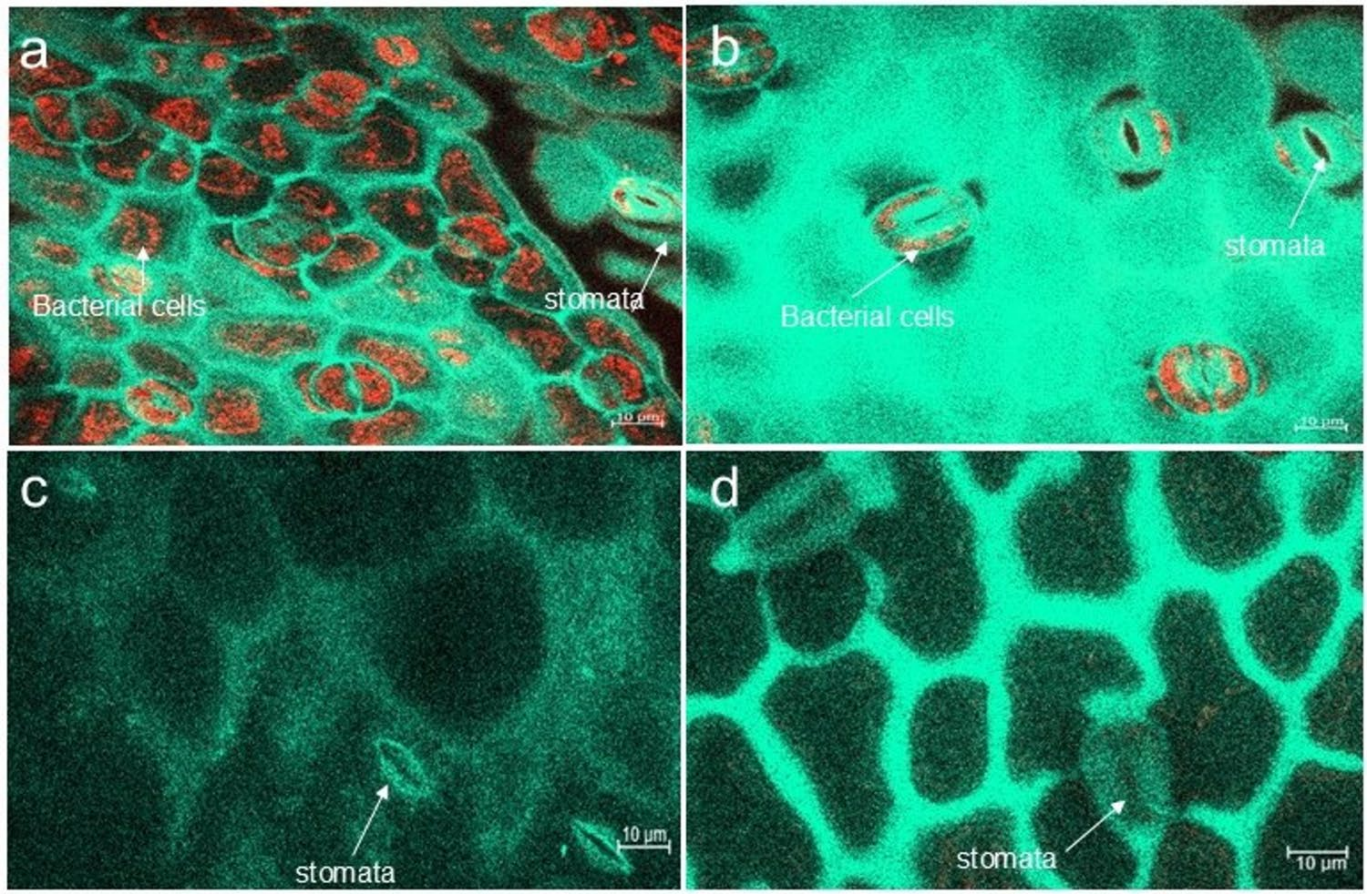
Confocal microscopy image showing the red fluorescence of the bacterial endophytes containing pLV-mCherry plasmids colonising the spinach leaf tissues. **a** leaf tissue (top side) **b** leaf tissue (below side) **c** Uninoculated plant leaf tissue (top) **d** uninoculated plant leaf tissue (below side)

The bacterial transformants were resistant to ampicillin and the presence of the pLV-mCherry plasmid in the bacterial cells was confirmed by gel electrophoresis of the RFP-mCherry gene (Supplementary Fig. 2) after enzyme digestion and PCR amplification. Microscopy revealed that the bacterial endophytes colonised the leaf tissues (Fig. 2). Confocal microscope transmitted light allowed for the visualisation of the plant tissues and the colonised bacterial transformants were detected by the red fluorescent light. The leaf tissues had a high colonisation frequency across the spinach plants (Fig. 2A, B) and the stomata were visible with evidence of colonisation establishment, while as expected, the control experiment, which had no mCherry-tagged bacterial inoculum showed no evidence of colonisation or fluorescence (Fig. 2C).

### Evaluation of Plant Growth-Promotion of Endophytic Bacteria

**Fig. 3:**
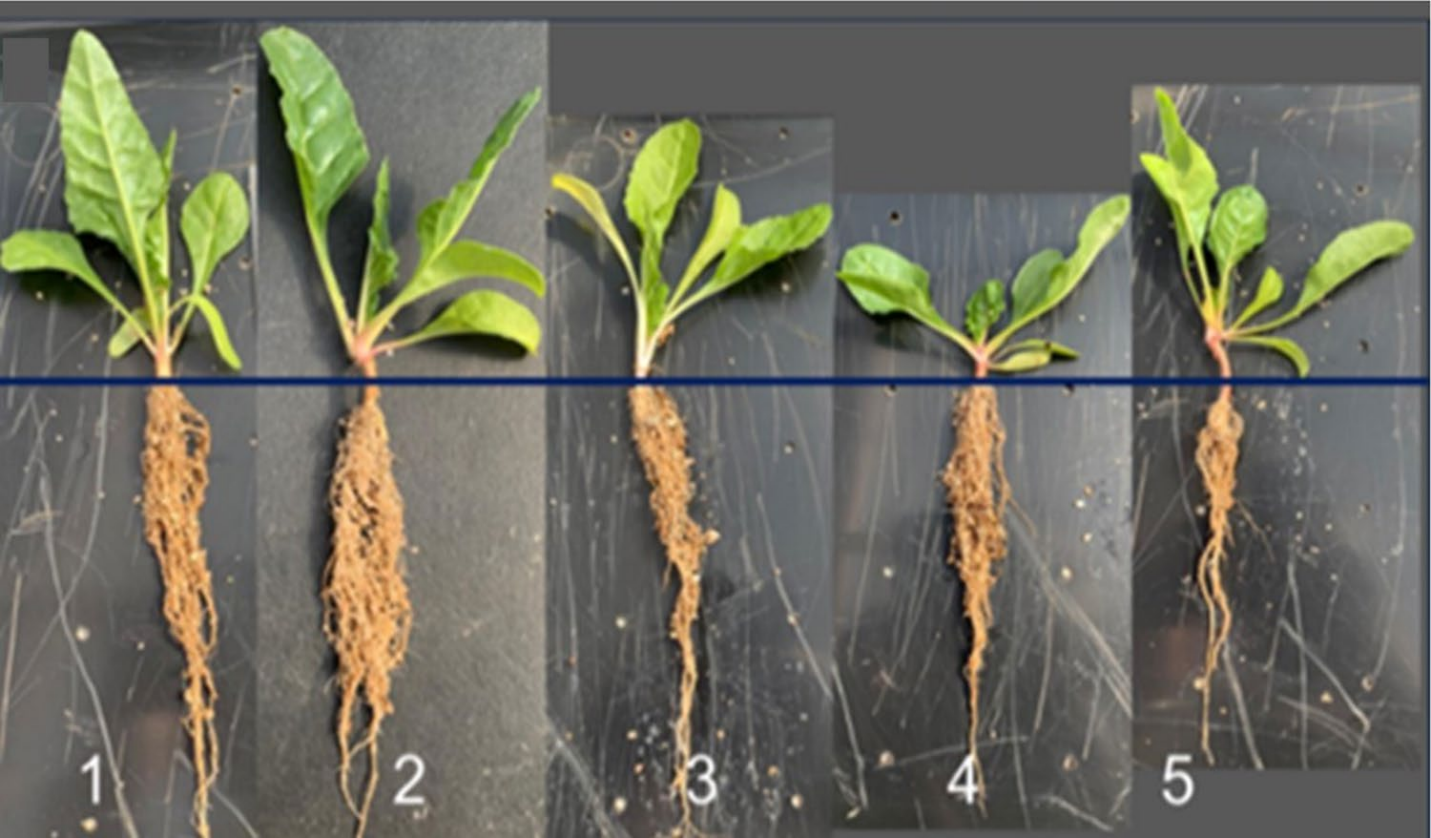
Plant growth-promoting ability of bacterial endophytes in a pot experiment. Treatments 1 = T2 (*Pseudomonas azotoformans)*, 2 = T3 (*Enterobacter bugandensis*), 3 = T4 (*Serratia marcescens)*, 4 = T1 (*Bacillus cereus*) and 5 = T0 (the control), The plants are arranged according to the root length

**Fig. 4:**
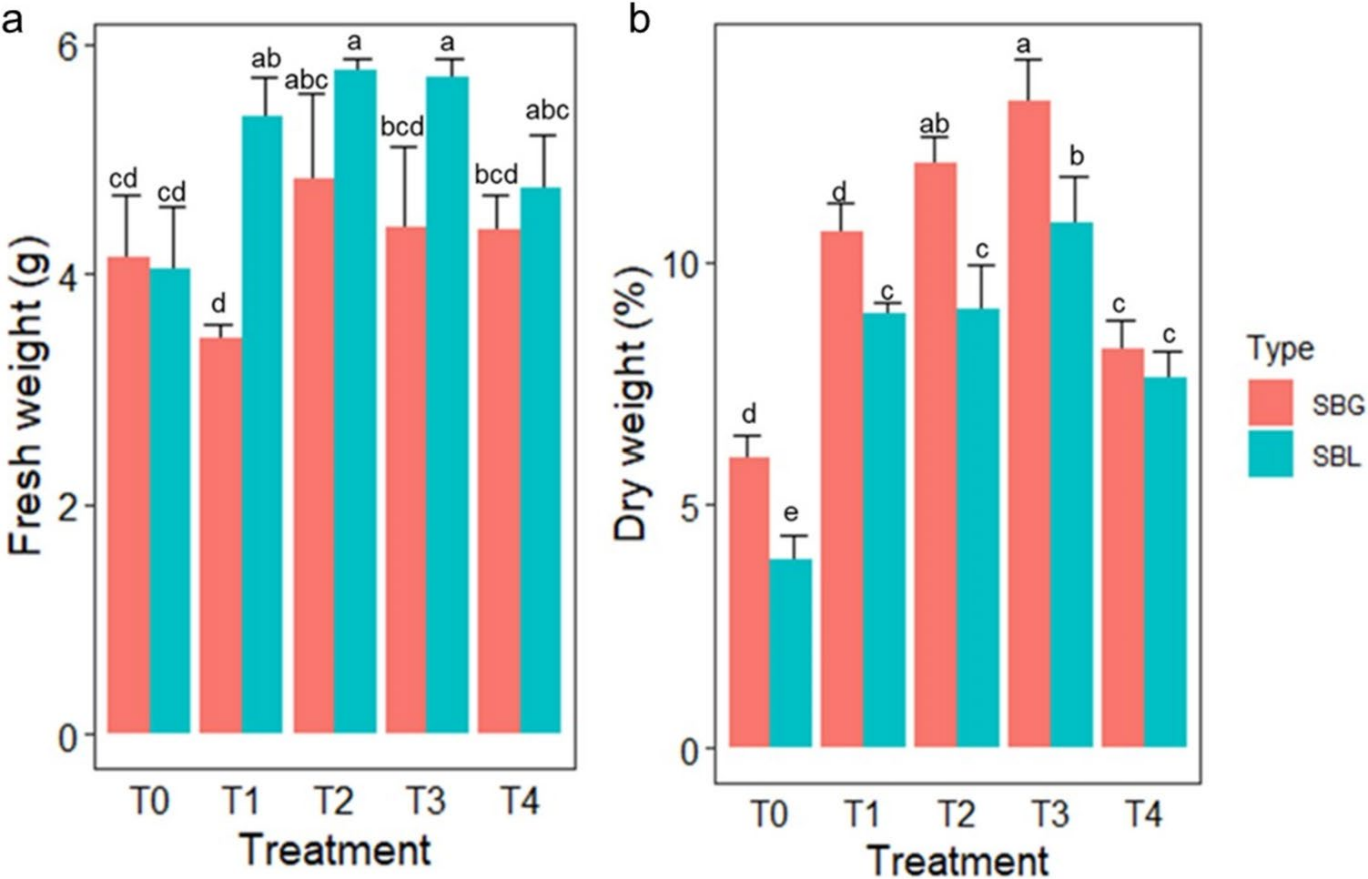
Growth effect of beneficial bacterial inoculum on spinach (Spinacia oleracea) leaf tissue, **a** Fresh weight and **b** dry weight. The treatments are T0; control, T1; *Bacillus cereus*, T2; *Pseudomonas azotoformans*, T3; *Enterobacter bugandensis* and T4; *Serratia marcescens*. BeetFordhook Giant (SBG) and Beet Lucullus (SBL) spinach plants. The plant’s fresh weight was measured in grams, while the dry weight was measured as a percentage. A two-way Analysis of Variance (ANOVA) was used to compare the statistically significant differences between the treatments using a *p*-value 0.05 threshold. The treatment was significant for fresh weight (df = 4, *p* = 1.27e-09) and dry weight (df = 4, 2e-16). Statistically significant treatments are represented with different alphabets

All the bacterial endophytes used in the pot experiment showed a higher ability to promote spinach growth, with varying growth effects when compared to the control (Fig. 3). Although the endophytic bacteria enhanced the growth parameters for both types of spinach, the plants treated with *P*. *azotoformans* showed considerable growth parameters and particularly enhanced the yield in SBG samples when compared with the SBL samples and the control (Table 4). The root length, shoot length, number of leaves (Table 4), fresh weight and dry weight (Fig. 4) were significantly (*P* < 0.05) higher in plants inoculated with* P*. *azotoformans* compared to the control. In the SBL samples, *B. cereus* caused a higher shoot length and number of leaves compared to the control and other treatments.

**Table 4:** Effect of bacterial inoculum on the growth parameters of spinach (*Spinacia oleracea*)

Treatment	Crop	Root length (mm)	Shoot length (mm)	No of leaves	Germination %	vigour index	Chlorophyll a	Chlorophyll b
Control	SBL	12.15 ± 0.62abc	7.13 ± 0.43ab	7 ± 0.82a	60 ± 7.07c	1156.8	28.77 ± 0.25a	15.11 ± 0.51d
*B. cereus*	SBL	14.03 ± 3.55ab	8.28 ± 0.99a	7 ± 1.00a	70 ± 4.08abc	1561.7	20.18 ± 1.20c	12.06 ± 2.83d
*P*. *azotoformans*	SBL	16.88 ± 3.40a	6.75 ± 0.99ab	6 ± 0.58a	80 ± 5.77a	1890.4	17.73 ± 1.41d	31.55 ± 1.68c
*E. bugandensis*	SBL	11.75 ± 1.23bc	7.83 ± 0.56a	6 ± 0.50a	75 ± 5.77ab	1468.5	26.86 ± 0.77a	52.11 ± 0.99a
*S. marcescens*	SBL	8.38 ± 1.11c	5.68 ± 0.46b	7 ± 0.58a	65 ± 7.07bc	913.9	23.65 ± 0.48b	42.97 ± 0.85b
Control	SBG	8.95 ± 0.47c	7.25 ± 0.87a	6 ± 0.58a	60 ± 7.07c	972	31.67 ± 0.42a	24.46 ± 0.32d
*B. cereus*	SBG	15.18 ± 1.58ab	6.78 ± 1.08a	6 ± 0.58a	70 ± 4.08abc	1537.2	30.51 ± 1.23ab	33.51 ± 1.84c
*P*. *azotoformans*	SBG	15.85 ± 3.31a	8.38 ± 1.11a	8 ± 1.50a	80 ± 5.77a	1938.4	24.37 ± 2.24d	51.89 ± 2.24a
*E. bugandensis*	SBG	11.5 ± 0.58bc	6.83 ± 1.11a	5 ± 0.50a	75 ± 5.77ab	1374.75	27.23 ± 0.41c	47.74 ± 0.41b
*S. marcescens*	SBG	9.3 ± 2.00c	6.7 ± 1.62a	6 ± 0.96a	65 ± 7.07bc	1040	28.52 ± 0.18bc	52.39 ± 0.18a

Data are presented as the mean ± standard deviation (*n* = 4). Values followed by different letters are significantly (*P* < 0.05) different within each column using a one-way analysis of variance with Tukey’s multiple comparison test. Each isolate represents a treatment. Chlorophyll was measured in mg/100 g

Interestingly, the total chlorophyll was highest (78.97 mg/100 g) among plants inoculated with *E. bugandensis* and *S. marcescens,* despite having lower growth parameters compared to *P*. *azotoformans* and *B. cereus*. The dry weight was observed to be significantly higher (13.3 ± 0.89%) in SBG plants (Fig. 4) inoculated with *E. bugandensis* followed by *P*. *azotoformans*, while the highest level of vigour index was observed in crops inoculated with *P*. *azotoformans* in comparison with other inoculants and uninoculated control plants. However, a considerable level of vigour index was also observed in plants inoculated with *E. bugandensis* and *B. cereus*. Bacterial endophytes caused a significant increase in plant roots and shoots and enhanced the dry weight and chlorophyll contents compared to the control. Seed sterilization did not affect the performance of the inoculum, as there were no noticeable differences between the plant yield parameters for sterilized and unsterilized plant seeds. No disease incidence was also noticed in any of the experiments.

## Discussion

This study investigated bacterial endophytes as a promising resource in the production of bioinoculants to enhance crop productivity. A major goal of sustainable agriculture is to improve crop productivity without any negative impact on the environment. To achieve this goal, there must be a reduction in the use of agrochemicals, which contribute to ecological damage [23]. Unfortunately, the rising demand for crops, especially vegetables, has further promoted the use of agrochemicals for increasing productivity, suggesting the need to intensify sustainable strategies to protect the agroecosystem. The plant growth-promoting endophytes (PGPEs) investigated in this study present a sustainable, cost-effective and eco-friendly strategy for improving crop productivity and alleviating ecological challenges [24]. The results of the virulence potential suggest that some bacterial endophytes could impact human health when vegetables are consumed raw. Plant microbiome plays a key role in influencing human gut microbiome assembly; thus, a healthy plant microbiome could promote a healthy human gut [11]. This is why it is critical to assess the virulence characteristics of microbial endophytes before they are harnessed for inoculant production.

### Antimicrobial, Antibiotic and Virulence Potential of Bacterial Endophytes

The antimicrobial profile of the tested bacterial endophytes reveals that endophytes are a promising source of antimicrobials and produce bioactive compounds of biotechnological importance. Bacterial endophytes are known to synthesize antibacterials such as 4-hydroxymellein, ketoconazole, polyketide, thiodiketopiperazine and organic extracts, which have antibacterial and antifungal activities against a spectrum of human pathogens [25, 26]. Similar to the observations in this study, endophytic bacteria such as *Pseudomonas* sp., *Bacillus licheniformis* and *Acinetobacter calcoaceticus* showed considerable antimicrobial activity against various human pathogens, including *Klebsiella pneumoniae*, *Salmonella typhi*, *Staphylococcus aureus*, *Streptococcus agalactiae* and *Proteus mirabilis* [26]. The endophytes *B. cereus, P. agglomerans,* and *E. bugandensis* with MIC 0.5 mg/mL are potential biocontrol agents. A study has reported similar endophytes with high inhibitory activity against human and plant pathogens [27], suggesting that endophytes are a promising resource for plant and human health management.

Antibiotic resistance (AR) in microbes, including bacterial endophytes, is a serious global threat to humanity. According to WHO, AR is among the top ten global public health challenges facing human beings [28]. Some of the bacterial endophytes are resistant to different classes of antibiotics tested, suggesting their potential impact on human health when consumed. A few of the endophytes are closely related to the genera that comprise pathogenic species such as *Salmonella*, *Mycobacterium, Pantoea, Enterobacter,* and *Serratia.* These pathogens inhabit spinach, lettuce, and rocket plants [29] and could cause diseases in the host. Leafy vegetables are rarely treated to eliminate associated microbes before being consumed raw or after minimal processing. This is the reason they are considered a major vehicle of foodborne illness outbreaks [30], suggesting it is crucial to identify the sources of pathogen contamination and transmission to better manage food safety risks associated with vegetable produce [31]. The use of organic manure and pesticides drives the increase in antibiotic resistance genes and bacteria in amended soil and cultivated crops [32]. Our finding shows that the tested endophytes are susceptible to many antibiotics, especially streptomycin, suggesting their low exposure to antibiotics in their natural environment. Moreover, endophytic bacteria reside inside plant tissues where they are relatively protected from external ecological factors such as antibiotic contaminants. This limits the evolution of antibiotic-resistant genes in such microbes and may suggest why *B. cereus, E. bugandensis*, *P. agglomerans*, *P. azotoformans* and *S. marcescens* were susceptible to most of the antibiotics. In addition, the bacteria may not grow well under laboratory conditions, making them more susceptible than they are in their natural habitat. However, practices promoting microbial pathogenicity and antibiotic resistance should be discontinued or at least reduced.

Vegetables are important for human health management, and their microbiome determines the microbial species that colonise and are integrated into the gut microbiome [33], suggesting human diet should not harbour pathogenic strains. Cytotoxicity potential varies among the tested endophytic species. This is due to the unique genetic makeup of each strain, which influences its ability to produce specific secondary metabolites such as alkaloids, polyketides and terpenoids with cytotoxic properties. Interestingly, the cytotoxicity assay suggested that some of the endophytes can potentially damage red blood cells, inhibit growth, and kill HuTu-80 cells. Bacterial endophytes with extremely high or high cytotoxicity (Table 2) may present a high health risk when consumed. A study has reported endophytic *Bacillus cereus* isolated from potatoes to show high cell toxicity in human and mammalian cells due to the production of cereulide, which causes foodborne illness in humans [34]. However, it is crucial to note that tissue or cell cultures are more susceptible to bacterial attack compared to live human intestinal cells, which have structured defence mechanisms against pathogens, thus necessitating further studies to ascertain the complete virulence nature of the organisms when consumed by humans. On the contrary, the cytotoxicity of endophytes may be harnessed in the treatment of cancer and other related diseases [35]. However, this is beyond the scope of this study, as the cytotoxicity, antimicrobial, and antibiotic susceptibility potential were used as part of the criteria for selecting efficient bacterial endophytes for the pot experiment to guarantee a healthy microbiome of the vegetables cultivated with the inoculant.

### Impact of Bacterial Endophyte Inoculum on Seed Germination

Seed germination is a crucial and delicate phase of the plant propagation cycle, ensuring plant adaptation and evolution. Improper germination strategies can cause loss of seed vigour, reduced plant density, low seedling growth and survival rates, and consequently, lower plant yield and nutritional quality [36]. It has been reported that inoculants of bacterial endophytes applied to seeds and seedlings enhanced the germination and yield of economically important crops such as maize, and wheat through improved nutrient availability and production of plant growth-promoting hormones. Endophytic *Bacillus selenatarsenatis* and *Achromobacter xylosoxidans,* which produced auxins, ACC deaminase and improved nitrogen availability, significantly enhanced the germination of *Artemisia annua* L. over the control, with the germination percentage in the range of 18–31% [37]. A similar result was observed in the present study, as the bacterial endophytes significantly (*P* < 0.05) promoted the germination of spinach seeds at a higher rate than the control. A commercial biostimulant containing *Bacillus* sp. significantly enhanced the germination of *Abelmoschus esculentus* seeds, with all germination parameters having higher values over the control [17]. This agrees with the results in this study, where the endophytes investigated, including *B. cereus, P. azotoformans, E. bugandensis* and *S. marcescens,* which can produce siderophores, auxins, ACC deaminase and fix nitrogen [2] enhanced the germination of spinach seeds with all germination parameters including FGP, MGT, GI, CVG and GRI significantly higher and improved when compared with the control, suggesting the great potential of endophytes in plant growth promotion (PGP). Genetic makeup plays a key role in plant physiological structure and activities, including germination processes such as DNA synthesis and repairs, and growth hormone production. Corroborating other studies, variations were observed in the germination parameters for the different types of spinach plants studied [38]. On the contrary, a study has reported no differences in the germination responses of different plant genotypes [39], suggesting that other factors, including seed dormancy, quality, treatment, and the different conditions of germination, may be highly identical [40]. 

### Plant Growth-Promoting Ability of Bacterial Endophytes

The results obtained from the pot experiments provided evidence of increased growth and yield of spinach by the different treatments of PGPE inoculants. Consistent with other studies, aboveground biomass (wet and dry weight) and root length were considered a measure of PGP in pot experiments [41]. Various studies have reported bacterial endophytes in the genera *Bacillus, Pseudomonas, Serratia, Acinetobacter, Klebsiella* sp. (PnB 10), and *Enterobacter* sp. (PnB 11) to promote the growth and health of different crops [42, 43]. Of all the endophytes, *Serratia* is poorly studied compared to *Pseudomonas* and *Bacillus,* which are widely used for bioinoculant production [44].

Furthermore, PGPEs employ several mechanisms, including N-fixation, phosphate solubilisation and phytohormone production to enhance plant growth [45]. Endophytic *Pseudomonas* and *Bacillus* are widely reported with high production of auxins and ACC deaminase [2, 37], known to promote root hair elongation. This may have contributed to the significant (*P* < 0.05) increase in the plant root length observed for treatments involving *P. azotoformans*. Similarly, the ability of endophytes to improve nutrient uptake and produce auxins, siderophores and hydrogen cyanide [2] may have influenced the high chlorophyll content observed in the treatments for *E. bugandensis* and *S. marcescens*. *Bacillus* usually colonise the primary root surface, including the zone of elongation, differentiation, and lateral root junction [12]. Root hairs are a major structure promoting endophyte colonisation, as shown in studies that used GFP-tagged endophytes, revealing that the GFP-tagged bacteria were localised with high density in the intercellular spaces of epidermal cells in the roots of tomato and rice plants [12, 46]. Similar observations were observed for the RFP-tagged PGPEs in this study, but in the leaf tissues (Fig. 2). Such systemic alteration in the plant after successful endophyte colonisation, which results in growth promotion, is better understood by functional genomic profiling. Inoculation of seeds used in this study allows for early association of PGPEs with the host plant from the point of germination, thereby promoting easy compatibility and establishment of the endophytes. Irrespective of the plant species investigated, endophytic *Methylobacterium suomiense* CBMB120 was transmitted to the aerial part of the host plant through seed inoculation [46], suggesting seed inoculation supports easy colonisation and growth promotion. However, a study did not find any significant difference in the effect of seed or foliar application of biofertiliser under waterlogging stress [47]. Though seed inoculation is widely used, dual inoculation (seed pre-sowing and soil after germination) combined with foliar application is recommended for the best results. 

The microscopy analysis revealed that the bacterial endophytes colonised the leaf tissues, similar to the observations reported in other studies [48, 49]. The microscopy showed a high density of the RFP-tagged bacterial endophytes around the stomata (Fig. 2). Stomata ensure optimum conditions for photosynthesis by facilitating the intake of carbon dioxide and release of oxygen and regulating transpiration by eliminating excess water vapour. Higher stomata density improved photosynthetic induction and biomass production in *Arabidopsis* under light fluctuation [50], suggesting the high bacterial density around the stomata may be contributing to stomata efficiency and photosynthesis, consequently, promoting a high yield of the host plant. The development of a confocal laser scanning microscope combined with fluorescence protein has brought new insight into the study of endophytes within their hosts. As observed in this study, these techniques revealed that bacterial inoculants colonise the internal tissues of crops to exhibit PGP [48, 49].

## Conclusion

The results of this study revealed that bacterial endophytes promote plant growth as indicated by the significant increase in yield parameters, including height, root length, chlorophyll content and biomass. The localisation of the endophytes within the plant tissues was successfully established using a confocal microscope. The microscopy study showed that endophytes are part of the plant’s physiological development. Endophytic *Pseudomonas*, *Enterobacter*, and *Bacillus*, which showed high growth parameters also had strong fluorescence colouration of the RFP-tagged bacterial endophytes. The virulence ability of the endophytes suggests their potential impact on human health and should be a major factor in inoculum selection. Overall, the bacterial endophytes present good resources in bioformulation for sustainable agriculture. However, future research should involve field experiments to establish the ability of the bacterial endophytes under uncontrolled environments. This will further improve the understanding of the ability of endophytes to compete and persist under natural conditions where more factors may influence their contribution to plant growth and development.

## Supplementary Information

Below is the link to the electronic supplementary material.

Supplementary file1 (DOCX 456 kb)
