# Supplementary material for: Evaluation of the Virulence and Plant Growth-Promoting Potential of Endophytic Bacteria for Improving Vegetable Production
Source: Curr Microbiol. 2025 Jul 10;82(8):374. doi: 10.1007/s00284-025-04356-1 (PMC12245939; doi:10.1007/s00284-025-04356-1)
Supplement: Supplementary file 1 — Supplementary file1 (DOCX 456 kb) [file 284_2025_4356_MOESM1_ESM.docx]

**Evaluation of the virulence and plant growth-promoting potential of endophytic bacteria for improving vegetable production.**

Adekunle Raimi^1^, Suranie Horn^2^, [Rialet Pieters](https://setac.onlinelibrary.wiley.com/authored-by/Pieters/Rialet)^1^, Rasheed Adeleke^,1 *^

^1^ Unit for Environmental Sciences and Management, North-West University, Potchefstroom, 2520, South Africa

^2^ Occupational Hygiene and Health Research Initiative (OHHRI), Faculty of Health Science,

North-West University, Private Bag X6001, Potchefstroom, South Africa

*Corresponding author

Email: rasheed.adeleke@nwu.ac.za, Phone no: +27 018 299 2495

**ORCID**: AR, 0000-0001-6437-6419; RA, 0000-0002-8974-422X.

Journal: Current Microbiology

**Supplementary information**

**Supplementary Text 1:**

**Crude extract of bacterial endophyte for minimum inhibitory concentration analysis**

100 mL of tryptic soy broth (TSB) (Biolab, USA) was inoculated with a single colony of the endophytic bacterial cells and incubated at 36 ℃ in the dark for 24 h. These cells were later transferred into a 400 mL sterile TSB and incubated for 96 h at 36 ℃ with shaking at 200 rpm. The OD was monitored at intervals by measuring a 10-fold dilution of 10 mL at 600 nm. At a constant OD, the culture was centrifuged at 10 000 x g for 20 min at 4 ℃ to separate the culture fluid from the bacterial cells. The supernatant was filtered through a 0.45 µm filter to obtain a cell-free culture filtrate. The mixture was neutralised with conc. hydrochloric acid (32%) and extracted with an equal volume of ethyl acetate and chloroform. The organic phase was dried in a vacuum drier. A 20 mg bacterial extract was dissolved in 1 mL of dimethyl sulfoxide to make a stock solution of 20 mg/mL, which was diluted using Mueller-Hinton broth to obtain different dilutions of 128 to 0.5 mg/mL. A 96-well plate was filled with 100 µL of the test organisms and 100 µL of the different concentrations of the crude extracts. Streptomycin and dimethyl sulfoxide were used as the positive and negative controls, respectively. The plates were incubated at 37 ℃ in a plate reader (Thermo Fisher, CA, USA) for 24 h. After incubation, 10 µL of 0.02% (w/v) resazurin sodium salt solution was added to each well as an indicator of bacterial cell growth. The plate was further incubated for 2 h at 37 ℃. A blue colouration indicates growth inhibition, while a pinkish colour indicates bacterial growth. The MIC value is the lowest concentration of the compound showing no visible growth.

**Supplementary Text 2**

**Maintenance and culture of HuTu-80 human intestinal cells (HTB-40™)**

HuTu-80 human intestinal cells (HTB-40™) obtained from the American Type Culture Collection (ATCC) (Manassas, VA, USA) were cultured in Dulbecco’s Modified Eagle’s Medium (DMEM) (Sigma, Darmstadt) supplemented with 10% foetal bovine serum (FBS) (Thermo Scientific, USA) and maintained in a humidified incubator, with 5% CO_2_ at 37°C. Cells were handled in a sterile laminar flow chamber disinfected with 70% ethanol and UV light for 15 min (Prinsloo et al., 2013). The HuTu-80 cells are duodenal adenocarcinoma cells and therefore never stop growing. Although these cancerous cells do not have all the constituents of primary cells, they are a good model for assessing the toxic potency of chemicals or organisms as a first-tier screening for cytotoxicity. To compensate for the effect of sterile broth, a second control was added at the same time as the bacteria.

**Supplementary Text 3**

**Seed germination testing and surface sterilisation**

Briefly, distilled water and filter papers (Whatman no 1) were sterilized by autoclaving. Seeds were surface sterilized following the method of McKinnon [1] using a combination of 5% v/v sodium hypochlorite and 0.05% v/v Tween 80 and 70% ethanol. Thirty seeds were placed on the filter papers moistened with equal volumes of distilled water in sterile Petri plates made in five replicates, placed in a container lightly closed with a plastic overlay to reduce moisture loss, and incubated at room temperature. Germination was confirmed by the radicle protruding at least 2 mm from the seed coat and germination was monitored daily to ascertain the normal growth of seedlings [2].

**Supplementary Text 4**

**Transformation of endophytic bacterial cells with mCherry insert plasmid**

Briefly, the competent cells, cuvette, sterile microcentrifuge tubes and SOC medium were placed on ice. 40 µL of the competent cells and 2 µg of the ligation mixture (plasmids coupled with mCherry insert) were placed into the microcentrifuge tube. This mixture was pipetted into a chilled 0.2 cm electroporation cuvette and inserted into a pulser controller attached to a Bio-Rad Gene Pulser machine (Bio-Rad Laboratories, England) set to 2.5 kV/cm and 25 µF and 200 Ω. The bacterial cells were removed from the cuvette and added to SOC medium to make 1000 µL and the mixture was then incubated at 37°C on a shaker set at 250 rpm for 1 h. 100 µL of the transformants were inoculated on a pre-warmed (37°C) LB agar supplemented with 100 µg/mL ampicillin and incubated at 37°C overnight. Positive transformants were picked and transferred into a new broth amended with 100 µg/mL ampicillin, incubated overnight at 37 °C with shaking at 50 rpm. The bacterial cells were stored at –80 °C after adding an equal volume of 40% glycerol.

**Supplementary Text 5**

**Evaluation of chlorophyll content**

A 0.5 g fresh leaf sample was macerated in a mortar with 20 mL of 80% acetone in distilled water and kept chilled over ice while processing under low luminosity. The mixture was centrifuged for 10 min at 3000 rpm in a microcentrifuge (Thermo Scientific, Ca, USA), and the supernatant was removed and made up to 50 mL using 80% acetone. The mixture was covered with aluminium foil and incubated for 30 min in the dark in a refrigerator. The solution was diluted appropriately, and the colour intensity of the green pigment was measured with a spectrophotometer (Thermo Scientific, Ca, USA) at 645 nm for chlorophyll a and 663 nm for chlorophyll b and 652 nm for total chlorophyll, while the results were expressed in mg/g.

**Supplementary Table**

**Supplementary Table 1:** Different classes of antibiotics for susceptibility testing

| Mechanisms of action | Antibiotics | Sub action |
| --- | --- | --- |
| Cell wall biosynthesis inhibition | Ampicillin (10 µg) |  |
|  | Amoxicillin (10 µg) |  |
|  | Vancomycin (30 µg) |  |
|  | Cefazolin (30 µg) |  |
| Bacterial protein synthesis inhibition | Gentamicin (10 µg) | 30S rRNA subunit inhibitor |
|  | Tetracycline (30 µg) |  |
|  | Streptomycin (10 µg) |  |
|  | Chloramphenicol (30 µg) | 50S rRNA subunit inhibitor |
|  | Erythromycin (15 µg) |  |
|  | Clindamycin (15 µg) |  |
| DNA synthesis inhibition | Ciprofloxacin (5 µg) |  |
|  |  |  |
| Folic acid synthesis inhibition | Trimethoprim (25 µg) |  |

**Supplementary Figure**


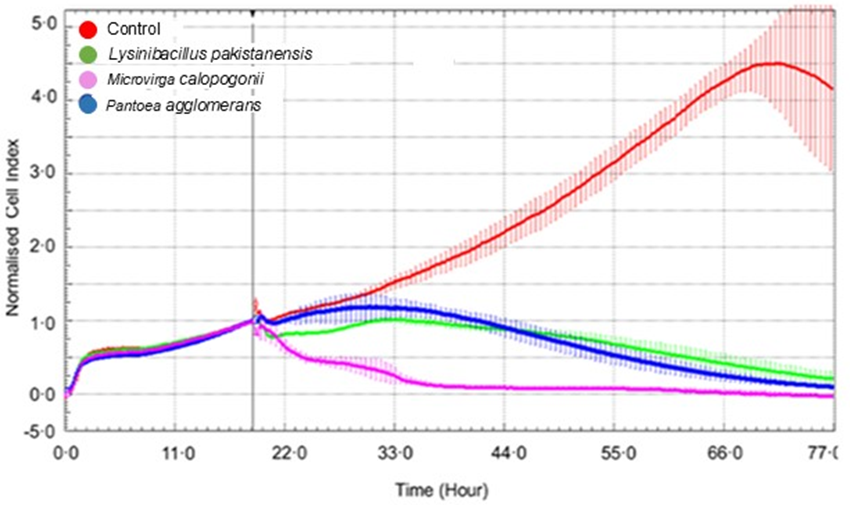


**Supplementary Fig. 1** Cytotoxicity of bacterial endophytes with gradual activity


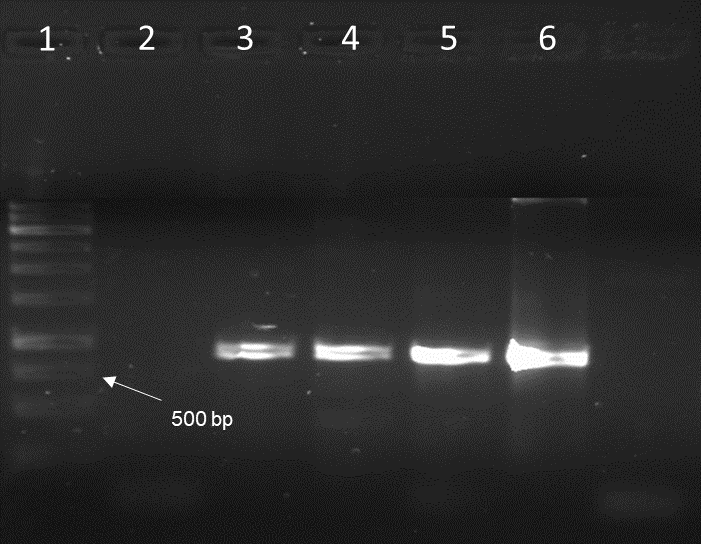


**Supplementary Fig. 2** Endophytic bacterial pLV-mCherry plasmid in gel electrophoresis. Lane 1; DNA ladder (1 Kb), 2; control and 3-6; mCherry gene

**References**

1. Mckinnon AC (2016) Plant tissue preparation for the detection of an endophytic fungus in planta. In: Travis R. Glare and Maria E. Moran-Diez (ed) Microbial-Based Biopesticides: Springer, New York, pp 167–173

2. Rao NK, Hanson J, Dulloo ME, et al (2006) Manual of Seed Handling in Genebanks. Handbooks for Genebanks No. 8. Bioversity International, Rome, Italy
